# Prediction of harmful variants on mitochondrial genes: Test of habitat‐dependent and demographic effects in a euryhaline fish

**DOI:** 10.1002/ece3.2989

**Published:** 2017-04-18

**Authors:** Anti Vasemägi, Janne Sulku, Matthieu Bruneaux, Olaf Thalmann, Hannu Mäkinen, Mikhail Ozerov

**Affiliations:** ^1^ Department of BiologyUniversity of TurkuTurkuFinland; ^2^ Department of AquacultureEstonian University of Life SciencesTartuEstonia; ^3^ Department of Biological and Environmental ScienceCentre of Excellence in Biological InteractionsUniversity of JyväskyläJyväskyläFinland; ^4^ Department of Pediatric Gastroenterology and Metabolic DiseasesPoznan University of Medical SciencesPoznanPoland

**Keywords:** adaptation, genetic load, mtDNA, nearly neutral theory of molecular evolution, selective constraint

## Abstract

Both effective population size and life history may influence the efficacy of purifying selection, but it remains unclear if the environment affects the accumulation of weakly deleterious nonsynonymous polymorphisms. We hypothesize that the reduced energetic cost of osmoregulation in brackish water habitat may cause relaxation of selective constraints at mitochondrial oxidative phosphorylation (OXPHOS) genes. To test this hypothesis, we analyzed 57 complete mitochondrial genomes of *Pungitius pungitius* collected from brackish and freshwater habitats. Based on inter‐ and intraspecific comparisons, we estimated that 84% and 68% of the nonsynonymous polymorphisms in the freshwater and brackish water populations, respectively, are weakly or moderately deleterious. Using in silico prediction tools (MutPred, SNAP2), we subsequently identified nonsynonymous polymorphisms with potentially harmful effect. Both prediction methods indicated that the functional effects of the fixed nonsynonymous substitutions between nine‐ and three‐spined stickleback were weaker than for polymorphisms within species, indicating that harmful nonsynonymous polymorphisms within populations rarely become fixed between species. No significant differences in mean estimated functional effects were identified between freshwater and brackish water nine‐spined stickleback to support the hypothesis that reduced osmoregulatory energy demand in the brackish water environment reduces the strength of purifying selection at OXPHOS genes. Instead, elevated frequency of nonsynonymous polymorphisms in the freshwater environment (*P*_n_ */P*_s_ = 0.549 vs. 0.283; Fisher's exact test *p* = .032) suggested that purifying selection is less efficient in small freshwater populations. This study shows the utility of in silico functional prediction tools in population genetic and evolutionary research in a nonmammalian vertebrate and demonstrates that mitochondrial energy production genes represent a promising system to characterize the demographic, life history and potential habitat‐dependent effects of segregating amino acid variants.

## 1 Introduction

Ever since interspecific DNA sequence data have become available, analyses of genetic variation between species have revealed the pervasive nature of purifying selection, that is, removal of deleterious mutations, acting on the majority of protein coding regions (Hughes, 2007). Concurrently, many studies have reported an excess of nonsynonymous variants within species, compared to estimates derived from between species comparisons, thus indicating that fitness reducing weakly deleterious mutations are not immediately eliminated from a population by natural selection (Hasegawa, Cao, & Yang, 1998; Nachman, Boyer, & Aquadro, 1994; Rand & Kann, 1996). This finding supports the nearly neutral theory, which contends that slightly deleterious mutations, whose effects lie between the selected and strictly neutral categories, play an important role in molecular evolution (Ohta, 1992). However, we know surprisingly little about how demographic history and ecological characteristics influence the efficacy and strength of purifying selection in natural populations (Elyashiv et al., 2010; Lohmueller, 2014).

The mitochondrial genome supplies a part of the protein machinery responsible for the production of most of the cell's energy (ATP) via oxidative phosphorylation (OXPHOS). Traditionally, mitochondrial DNA (mtDNA) has been used as a neutral genetic marker in phylogenetic and phylogeographic studies (Avise, 2001). However, it is widely acknowledged that mtDNA is not functionally neutral and mtDNA diversity may be influenced by selective sweeps or/and background selection, rather than accurately reflecting population history and demography (e.g., Bazin, Glémin, & Galtier, 2006; Galtier, Nabholz, Glémin, & Hurst, 2009; Sloan, Havird, & Sharbrough, 2016). Consistent with its pivotal role in energy production, the predominant evolutionary force that shapes mtDNA variability across taxa is purifying selection (Rand, 2001); however, growing evidence also suggests that mitochondrial OXPHOS genes have experienced non‐negligible levels of positive selection (James, Piganeau, & Eyre‐Walker, 2016). The high mutation rate of mtDNA combined with the lack of an efficient means to remove slightly deleterious mutations due to the absence of recombination causes an accumulation of harmful substitutions, particularly when effective population size (*N*_e_) is low (Lynch, 1996). Thus, weakly or moderately deleterious mutations are expected to be more frequent in populations with small *N*_e_ (Kimura, 1962; Ohta, 1992) and/or in populations in which selection has been relaxed (Björnerfeldt, Webster, & Vilà, 2006; Hughes, 2013). For example, large mammals with lower *N*_e_ have been shown to accumulate nonsynonymous substitutions at mitochondrial genes at a higher rate than small mammals with higher *N*_e_, suggesting that the efficiency of purifying selection depends on *N*_e_ (Popadin, Polishchuk, Mamirova, Knorre, & Gunbin, 2007). Alternatively, relaxation of selective constraints (i.e., a shift in the selection coefficient towards zero) can lead to a faster accumulation of potentially harmful nonsynonymous nucleotide substitutions, as is evident from comparisons of mitochondrial OXPHOS genes between flightless and flying birds (Shen, Shi, Sun, & Zhang, 2009). Similarly, comparisons between high‐performance swimmers, such as tunas, and sedentary fish revealed relaxation of purifying selection on mitochondrial OXPHOS genes among the latter, indicating that high‐performance swimmers have lower tolerance for nonsynonymous, potentially disadvantageous substitutions in mtDNA (Strohm et al. 2015; Zhang & Broughton, 2015).

In contrast to terrestrial species, aquatic organisms face unique osmoregulatory challenges to maintain their optimal body fluid homeostasis at various salinities. To compensate for passive water loss, marine teleosts consume seawater and actively secrete salt, whereas freshwater fish balance the passive water gain by producing diluted urine and actively absorbing salt through their gills. Maintaining body fluid homeostasis is a complex and energetically expensive physiological process, and it is estimated that 20% to over 50% of the total energy budget in fish is used for osmoregulation (reviewed by Boeuf & Payan, 2001). However, the energetic cost of osmoregulation in brackish water is expected to be lower than in saltwater or freshwater, because the osmotic gradient between blood and water is reduced. These energy savings are sufficient to increase the growth rate of the fish and almost all marine fish grow faster at lower salinities, whereas freshwater fish grow faster at higher salinities (Boeuf & Payan, 2001). Therefore, we hypothesize that the reduced energetic cost of osmoregulation in brackish water habitat may cause relaxation of selective constraints at mitochondrial OXPHOS genes.

To test whether the efficacy of purifying selection at mitochondrial genes is affected by reduced *N*_e_ or if the decreased osmoregulatory energy demand in brackish water reduces the intensity of purifying selection, we analyzed complete mitochondrial genomes of nine‐spined stickleback (*Pungitius pungitius*) collected from both freshwater (populations with a relatively low *N*_e_) and brackish water (populations with a relatively high *N*_e_) environment in the Baltic Sea region. In order to infer the role of purifying selection on the mitochondrial OXPHOS genes, we evaluated the proportion of nonsynonymous and synonymous polymorphisms and substitutions within and between species, respectively, and used recently developed in silico tools that predict the harmful effect of nonsynonymous mutations (Hecht, Bromberg, & Rost, 2015; Li et al., 2009). The latter approach is commonly used in medical genetics, but has only very recently been applied in the evolutionary genetic framework (Barson et al., 2015; Soares et al., 2013). We predict that small freshwater populations will exhibit higher proportion of nonsynonymous polymorphisms with harmful effect if the difference in *N*_e_ between freshwater and brackish water fish is the main driver of the efficacy of purifying selection in these populations. In contrast, we expect higher proportion of nonsynonymous polymorphisms and higher frequency of harmful nonsynonymous substitutions in brackish water populations if the mtDNA OXPHOS genes are under relaxed selective constraints due to a reduced osmoregulation energetic cost in the brackish water environment.

## 2 Materials and Methods

### 2.1 Sampling, mitogenome sequencing, and phylogenetic and microsatellite analyses

**Figure 1:**
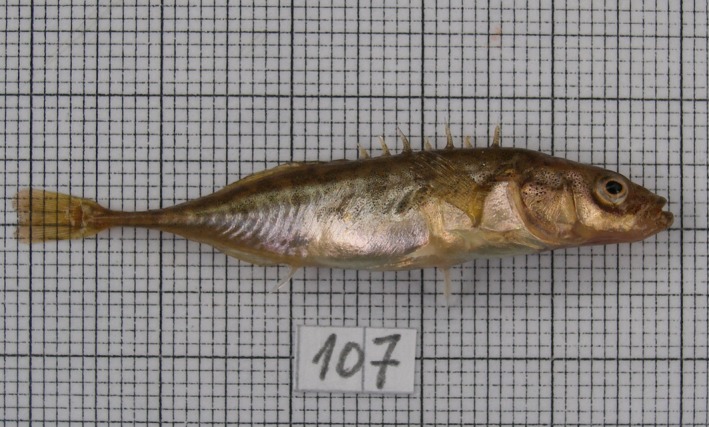
An individual of nine‐spined stickleback (*Pungitius pungitius*) collected from freshwater lake (Roosna‐Alliku). Photograph taken by Matthieu Bruneaux

**Figure 2:**
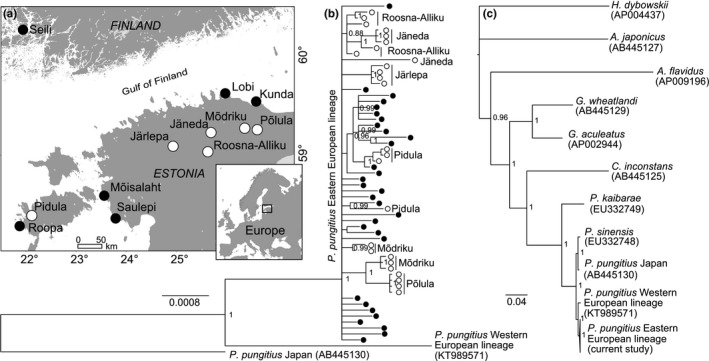
Map showing (a) the sampling sites, (b) *P. pungitius,* and (c) interspecific phylogenies inferred from the Bayesian analyses. Black and white circles indicate the brackish and freshwater environments, respectively. Posterior probabilities > 0.8 are shown

In total, 478 nine‐spined stickleback individuals (Figure 1) belonging to the Eastern European lineage (Wang, Shikano, Persat, & Merilä, 2015) were collected from six brackish water (Baltic Sea, salinity 4‰–6‰, *n* = 230) and six freshwater locations within the Baltic basin (*n* = 248, Figure 2a). The individuals from freshwater habitats were caught from small lakes, ponds (0.01–0.47 km^2^), or from tiny streams/ditches inflowing or outflowing from these ponds using standard electrofishing equipment. The individuals from the brackish water habitats were sampled in the Baltic Sea near the shores of the Gulfs of Finland and Riga and from the Archipelago Sea using beach seine.

In total, 57 nine‐spined sticklebacks from freshwater (*n* = 26) and brackish water (*n* = 31) habitats were used for complete mitochondrial genome sequencing (Table 1). Individual mitogenomes were amplified using long‐range PCR with two sets of primer pairs (Table S1). Sequencing on the Ion PGM™ using Ion 314™ and Ion 316™ chips (Life Technologies, Germany) resulted in complete mitochondrial genomes with high coverage (Table S2). The reads were mapped to the reference *P. pungitius* sequence (AB445130) using MIA (Green et al., 2008). All consensus sequences were aligned with MAFFT (Katoh, Misawa, Kuma, & Miyata, 2002), and the alignment was checked manually in BioEdit v.7.2.5 (Hall, 1998). Additionally, mitochondrial genomes from *P. pungitius* from Japan (AB445130) and Western Europe (KT989571), *P. sinensis* (EU332748), *P. kaibarae* (EU332749), *Culaea inconstans* (AB445125), *Gasterosteus wheatlandi* (AB445129), *G. aculeatus* (AP002944), *Hypoptychus dybowskii* (AP004437), *Aulichthys japonicus* (AB445127), and *Aulorhynchus flavidus* (AP009196) were obtained from GenBank to infer phylogenetic relationships. The evolutionary relationships were analyzed under Bayesian inference using MrBayes v.3.1.2 (Ronquist et al., 2012) based on the complete mitogenome, except for the D‐loop because of incomplete sequence data in some species. The posterior probabilities of the clusters were estimated using two independent runs of four Markov chains with 10^6^ generations. Every 100 generations were sampled, and the first 25% of the samples were excluded as burn‐in. The phylogenetic relationships were visualized using Figtree v.1.4.2 (http://tree.bio.ed.ac.uk/software/figtree/). Basic descriptive sequence statistics for the complete mitogenome and mitochondrial OXPHOS genes were calculated using DnaSP v.5.1 (Librado & Rozas, 2009) and PopGenome v.2.6.1 (Pfeifer, Wittelsburger, Ramos‐Onsins, & Lercher, 2014) in R v.3.2.5 (R Core Team 2016). The individual samples from six freshwater (*n* = 26) and six brackish water (*n* = 31) populations were pooled for further statistical analyses to obtain separate mtDNA diversity estimates for freshwater and brackish water habitats.

**Table 1:** Summary statistics for 57 nine‐spined stickleback mitogenomes

Population	*n*	*S*_T_	*S*_G_	*P*_n_	*P*_s_	*P*_n_/*P*_s_	*d*_N_/*d*_S_	*N*_h_	*h* (±*SD*)	π (±*SD*)	θ (±*SD*)
Freshwater											
Jäneda	4	32	24	7	17	0.412	0.141	3	0.833 (±0.222)	0.00098 (±0.00050)	0.00107 (±0.00019)
Järlepa	4	5	3	1	2	0.500	0.171	3	0.833 (±0.222)	0.00015 (±0.00005)	0.00017 (±0.00007)
Mõdriku	5	23	16	7	9	0.778	0.267	5	1.000 (±0.126)	0.00052 (±0.00013)	0.00045 (±0.00012)
Põlula	4	2	1	0	1	0.000	0.000	3	0.833 (±0.222)	0.00007 (±0.00002)	0.00007 (±0.00005)
Pidula	4	22	18	9	9	1.000	0.343	2	0.667 (±0.314)	0.00090 (±0.00042)	0.00090 (±0.00019)
Roosna‐Alliku	5	21	12	4	8	0.500	0.171	5	1.000 (±0.126)	0.00055 (±0.00011)	0.00062 (±0.00013)
Total/average	26	116	79	28	51	0.549	0.188	19	0.969 (±0.020)	0.00089 (±0.00009)	0.00140 (±0.00016)
Brackish water											
Kunda	9	79	59	14	45	0.311	0.107	9	1.000 (±0.052)	0.00111 (±0.00011)	0.00180 (±0.00021)
Lobi	7	61	48	9	39	0.231	0.079	7	1.000 (±0.076)	0.00110 (±0.00017)	0.00148 (±0.00020)
Mõisalaht	3	19	17	3	14	0.214	0.074	3	1.000 (±0.272)	0.00078 (±0.00022)	0.00078 (±0.00018)
Roopa	9	85	67	16	51	0.314	0.108	9	1.000 (±0.052)	0.00114 (±0.00010)	0.00181 (±0.00020)
Saulepi	2	11	8	2	6	0.333	0.115	2	1.000 (±0.500)	0.00067 (±0.00034)	0.00067 (±0.00020)
Seili	1	n/a	n/a	n/a	n/a	n/a	n/a	n/a	n/a	n/a	n/a
Total/average	31	239	186	41	145	0.283	0.097	31	1.000 (±0.008)	0.00099 (±0.00007)	0.00353 (±0.00025)

*S*, the number of polymorphisms based on the complete mitogenome sequence (*S*_T_) and 13 mitochondrial genes (*S*_G_), *P*_n_, the number of nonsynonymous polymorphisms, *P*_s_, the number of synonymous polymorphisms; *d*_N_/*d*_S_ ratio; *N*_h_, the number of haplotypes, *h*, haplotype diversity, π, nucleotide diversity, and θ per site (based on the complete mitogenome sequence).

In addition to mitochondrial genome sequencing, 462 individuals from five freshwater and five brackish water populations were initially screened at 12 microsatellite loci (Bruneaux et al., 2014; Table 2). However, three loci were removed from the final data analysis because of significant deviations from the Hardy–Weinberg equilibrium (*p* < .001). Altogether, 48 individuals were genotyped from each location with the exception of the Saulepi population, where 30 samples were genotyped (Table 2). Basic population genetic statistics, such as the expected (*H*_E_), observed heterozygosity (*H*_O_), and allelic richness (*A*_R_), were estimated as implemented in FSTAT v.2.9.3.2 (Goudet, 1995). Effective population sizes (θ = 4*N*_e_ × μ, where *N*_e_ is effective population size, μ is the mutation rate per site per generation) were estimated based on multilocus microsatellite genotypes using coalescent‐based Bayesian inference implemented in Migrate 3.6.11 (Beerli & Felsenstein, 2001). Mutation‐scaled population size was estimated using a continuous Brownian motion model with a Markov chain Monte Carlo (MCMC) repetition of combination of five long chains of 500,000 steps each with 10,000 burn‐in. *N*_e_ estimates were derived assuming a microsatellite μ = 5 × 10^−4^ (Estoup & Angers, 1998). As a rough indicator of the loss of heterozygosity in freshwater populations, we calculated the inbreeding coefficient *F* = (*H*_E_ brackish − *H*_E_ freshwater)/*H*_E_ brackish. We assumed that the genetic diversity in the brackish water populations mirrored the diversity in the early phases of freshwater colonization. In other words, the brackish water populations were the source from which the freshwater populations were derived. Thus, the *F* parameter describes the loss of diversity due to genetic drift since the colonization of freshwater after the last glaciation.

**Table 2:** Estimated diversity indices based on nine microsatellite loci

Population	*n*	*H*_E_ (±*SD*)	*H*_O_ (±*SD*)	*NA* (±*SD*)	*A*_R_	*F*_IS_	*N*_e_ (95% CI)	*F*
Freshwater								
Jäneda	48	0.53 (±0.09)	0.49 (±0.02)	5.78 (±4.84)	5.20	0.075	130 (0–273)	0.25
Mõdriku	48	0.32 (±0.11)	0.33 (±0.02)	3.33 (±3.35)	3.05	−0.018	137 (0–280)	0.54
Põlula	48	0.42 (±0.09)	0.44 (±0.02)	3.56 (±2.51)	3.33	−0.037	63 (0–173)	0.40
Pidula	48	0.60 (±0.08)	0.56 (±0.02)	6.78 (±4.84)	6.10	0.067	110 (0–247)	0.15
Roosna‐Alliku	48	0.57 (±0.08)	0.51 (±0.02)	6.33 (±5.29)	5.49	0.114	70 (0–193)	0.18
Total/average	240	0.49 (±0.09)	0.47 (±0.02)	5.16 (±4.17)	4.63	0.040		0.30
Brackish water								
Kunda	48	0.72 (±0.05)	0.72 (±0.02)	10.67 (±10.00)	8.85	−0.001	503 (273–707)	
Lobi	48	0.70 (±0.06)	0.65 (±0.02)	11.78 (±10.54)	9.56	0.075	477 (227–693)	
Mõisalaht	48	0.70 (±0.05)	0.68 (±0.02)	11.89 (±10.61)	9.49	0.036	390 (180–580)	
Roopa	48	0.70 (±0.05)	0.70 (±0.02)	10.56 (±9.02)	8.60	−0.001	397 (173–600)	
Saulepi	30	0.70 (±0.06)	0.67 (±0.03)	9.89 (±8.58)	9.55	0.038	557 (333–767)	
Total/average	222	0.70 (±0.05)	0.68 (±0.02)	10.96 (±9.75)	9.21	0.029		

*H*_E_, expected and *H*_O_, observed heterozygosity, *NA*, mean number of alleles, *A*_R_, allelic richness, *F*_IS_, inbreeding coefficient, *N*_e_, effective population size, *F*, loss of genetic diversity in freshwater populations.

### 2.2 Estimation of the proportion of harmful nonsynonymous polymorphisms

We estimated the proportion of harmful polymorphisms at OXPHOS genes in the *P. pungitius* mitochondrial genome as described in Subramanian (2011, 2012). The fraction of weakly or moderately deleterious polymorphisms (δ) was obtained by comparing the proportion of nonsynonymous (*P*_n_) and synonymous (*P*_s_) polymorphisms within *P. pungitius* with the ratio of nonsynonymous (*D*_n_) and synonymous (*D*_s_) substitutions between the nine‐ and three‐spine sticklebacks (*G. aculeatus*) using the following formula: δ = 1 − (*D*_n_ *P*_s_/*D*_s_ *P*_n_). To obtain confidence intervals for δ, we used a bootstrap procedure by resampling both within species and fixed between‐species substitutions (10,000 replications).

### 2.3 In silico prediction of the functional effect of nonsynonymous variants

We employed two prediction tools as implemented in the programs MutPred v.1.2 (Li et al., 2009) and SNAP2 (Hecht et al., 2015) to infer the functional impact of nonsynonymous variants in OXPHOS genes. As the prediction of functional effect of the same amino acid variant may vary substantially among methods, in practice the use of different in silico prediction tools has been recommended (e.g., Thusberg et al. 2011). Both prediction methods have shown high accuracy compared to other prediction tools (Hecht et al., 2015; Thusberg et al. 2011; Walters‐Sen et al. 2015) and demonstrated good performance on nonhuman data, including mammals (Hecht et al., 2015; Soares et al., 2013) and bacteria (Lind, Arvidsson, Berg, & Andersson, 2017). The method implemented in MutPred represents a random forest‐based classification tool that utilizes 14 different structural and functional protein properties, including helical propensity or loss of a phosphorylation site, and evolutionary conservation data to predict whether an amino acid variant has a phenotypic effect (Li et al., 2009). The MutPred pathogenicity score ranges from zero to one, with higher scores indicating a greater likelihood that the amino acid variation has a potentially harmful effect. Second, a neural network‐based tool implemented in SNAP2 was used to predict the functional impact of nonsynonymous substitutions (Hecht et al., 2015). Similar to MutPred, SNAP2 combines various parameters, including evolutionary (taken from an automatically generated multiple sequence alignment), structural (e.g., predicted secondary structure and solvent accessibility) and functional (e.g., amino acid properties and predicted disordered regions) information. The SNAP2 score ranges from −100 to 100, with higher scores indicating a greater probability of the specific mutation to alter the native protein function. Because the prediction of the functional effect of amino acid changes depends on the direction (i.e., the predicted effect of Gly to Asp does not equal an Asp to Gly change), nonsynonymous polymorphisms with a lower allele frequency within *P. pungitius* were classified as derived alleles. The ancestral status of more frequent amino acid variants was confirmed using phylogenetic information (Figure 2c). For interspecific comparisons between the nine‐ and three‐spined sticklebacks, we determined the derived and ancestral amino acid variants using the ANCESCON software (Cai, Pei, & Grishin, 2004). For the reconstruction of ancestral protein sequences, we used a Bayesian phylogenetic tree based on the mitogenomes of nine species (*P. pungitius, P. kaibarae, P. sinensis, C. inconstans*,* G. wheatlandi, G. aculeatus, H. dybowskii*,* A. japonicus,* and *A. flavidus*) constructed as described above. When the estimated ancestral amino acid at a particular position was identical to observations in either the nine‐ or three‐spined stickleback, we quantified the functional effect of the amino acid change from the ancestral to the derived state. By contrast, when the ancestral amino acid at a particular position (11 cases) was different in both the nine‐ and three‐spined sticklebacks, we estimated the functional effect of the amino acid change separately for both species. The distributions of the functional scores for both the inter‐ and intraspecific comparisons were visualized using the Gaussian kernel density estimator with default bandwidth as implemented in the R package stats v.3.2.5 (R Core Team 2016).

### 2.4 Estimation of the strength of purifying selection in relation to predicted effects

To assess how the strength of purifying selection varied with the predicted functional scores, we divided the distribution of the observed MutPred and SNAP2 scores by the distribution of the scores for all potential amino acid substitutions as described in Pereira, Soares, Radivojac, Li, and Samuels (2011) using an exponential function. We calculated the MutPred and SNAP2 scores for all potential amino acid variants for both the nine‐ and three‐spined sticklebacks at 13 mitochondrial OXPHOS genes. Because the distribution of functional scores for both species was almost identical, only the potential amino acid variant scores in *P. pungitius* were used to visualize the relative strength of purifying selection in relation to the predicted functional impact.

## 3 Results

### 3.1 Genetic diversity and phylogeny

Fifty‐seven complete mitochondrial *P. pungitius* genomes contained a total of 330 variable sites (brackish water, 31 genomes with 236 variable sites; freshwater, 26 genomes with 115 variable sites; Table S3). The phylogenetic analysis revealed a deep divergence between the western and eastern European lineages of *P. pungitius*, consistent with earlier reports (Wang et al., 2015). The general topology of the phylogenetic tree among the nine species was similar to an earlier study based on full mitochondrial genomes and 11 nuclear genes (Kawahara, Miya, Mabuchi, Near, & Nishida, 2009). The tree revealed a very low degree of divergence among the sequenced *P. pungitius* samples. However, weak structuring of freshwater individuals in accordance with their respective population of origin suggests that these variants became fixed during or after the colonization of freshwater habitat (Figure 2b). As expected, freshwater populations showed lower genetic diversity (15%–54% loss of heterozygosity) compared to brackish water populations based on nuclear microsatellite markers (Table 2; Mann–Whitney *U*‐test, *H*_E_ *p* = .010, *H*_O_ and *A*_R_ both *p* = .012) reflecting smaller *N*_e_ in the former (Table 2; Mann–Whitney *U*‐test, *p* = .012). On the other hand, the loss of genetic diversity at mtDNA in freshwater was less pronounced at population level (Table 1; Mann–Whitney *U*‐test, π *p* = .056 and θ *p* = .055). Despite the lower genetic variability and smaller *N*_e_ of freshwater populations, multiple distinct mitochondrial haplotypes segregated within several freshwater nine‐spined stickleback populations (Tables 2 and S4).

### 3.2 Synonymous and nonsynonymous polymorphisms

We detected 145 synonymous and 41 nonsynonymous polymorphisms in *P. pungitius* in the brackish water habitat and 51 and 28 synonymous and nonsynonymous polymorphisms, respectively, in the freshwater habitat. Thus, the proportion of nonsynonymous polymorphisms in the freshwater populations was almost two times higher compared to brackish water populations (*P*_n_ */P*_s_ = 0.549 vs. 0.283; Fisher's exact test *p* = .032), suggesting that purifying selection might be less efficient in freshwater populations with lower *N*_e_ values (Tables 1 and S3). Furthermore, resampling of the polymorphisms observed in the brackish water habitat indicated that the elevated frequency of nonsynonymous variants in the freshwater environment was not caused by the small sample size and lower variability (one‐sided permutation test, *p* = .0047). Similarly, estimated *d*_N_/*d*_S_ ratios across 13 OXPHOS genes in the nine‐spine stickleback were almost twice as large in the freshwater (*d*_N_/*d*_S_ = 0.188) compared with the brackish water habitat (*d*_N_/*d*_S_ = 0.097; Tables 1 and S3). However, none of the individual mitochondrial OXPHOS genes reached statistical significance, with *ND4* showing the largest difference in *P*_n_ */P*_s_ between the two habitats (*P*_n_ */P*_s_ = 1 and 0.273 in the freshwater and brackish water habitats, respectively; Fisher's exact test *p* = .078; Table S3). In contrast to the within‐species analysis, numerous (*n* = 1565) synonymous substitutions fixed between the nine‐ and three‐spine sticklebacks were observed, whereas only a small proportion (*n* = 141) of nonsynonymous substitutions were fixed between the two stickleback species (*P*_n_ */P*_s_ = 0.090, *d*_N_/*d*_S_ = 0.031). By assuming that the ratio of nonsynonymous to synonymous polymorphisms within species is similar to the ratio of nonsynonymous to synonymous substitutions between species under neutral evolution (McDonald & Kreitman, 1991), we estimated that 84% (95% CI: 72%–90%) and 68% (95% CI: 52%–78%) of the nonsynonymous polymorphisms in the freshwater and in the brackish water *P. pungitius* populations, respectively, were weakly or moderately deleterious. However, because the freshwater populations showed lower genetic diversity, the estimated number of harmful polymorphisms in the mitochondrial OXPHOS genes per individual (i.e., the mutation load) was identical in both habitats (on average 0.9 slightly deleterious mutations per mitochondrial genome).

### 3.3 Prediction of the functional effect of nonsynonymous variants

**Figure 3:**
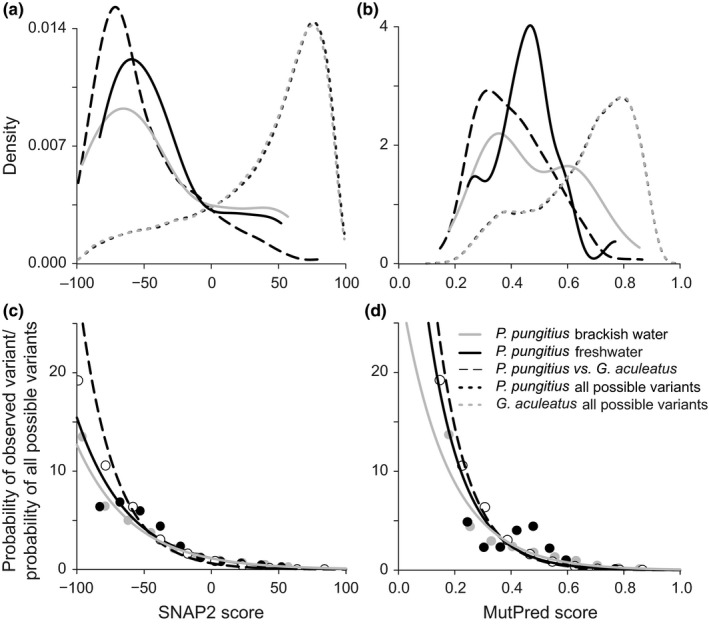
The distribution of the predicted effects of nonsynonymous substitutions (a, b) with higher values indicating an increased likelihood of functional effect. The estimated exponential selection function (c, d) based on the SNAP2 and MutPred scores. Gray, black, and open dots correspond to the *P. pungitius* brackish water, *P. pungitius* freshwater, and *P. pungitius* vs. *G. aculeatus* comparisons, respectively

The distribution of the SNAP2 and MutPred scores for polymorphisms in *P. pungitius* was strongly shifted toward low values compared with the predicted effects of all potential variants, indicating a pervasive role of purifying selection on the mitochondrial genome (Figure 3a,b; all Mann–Whitney *U*‐tests *p* < 1.3 × 10^−9^). In total, 15 and 13 of 65 amino acid variants in *P. pungitius* showed ≥0.6 and >0 functional scores for MutPred and SNAP2, respectively, implying potential functional effect (Table S4). Both tools predicted consistent effects for seven nonsynonymous polymorphisms, while the predictions between the two methods were inconsistent for 14 amino acid variants. Nevertheless, the SNAP2 and MutPred scores were correlated for all within‐species comparisons (*P. pungitius* observed variants, Spearman *r*_S_ = .35, *p* = 3.9 × 10^−3^; *P. pungitius* all possible variants, *r*_S_ = .57, *p* = 2.2 × 10^−16^; *G. aculeatus* all possible variants, *r*_S_ = .58, *p* = 2.2 × 10^−16^) but not for the interspecific comparison between *P. pungitius* vs. *G. aculeatus* (*r*_S_ = .13, *p* = .12).

The majority of nonsynonymous polymorphisms occurred at low frequencies (average minor allele frequency MAF = 0.026; range 0.015–0.108). The distribution of the functional scores calculated for singletons was similar to the one observed for variants that occurred at a higher frequency (found in ≥2 individuals) for both SNAP2 and MutPred (both Mann–Whitney *U*‐tests *p* > .05; Table S4). However, singletons were predicted to have a functional effect more often than frequent variants when the SNAP2 and Mutpred scores were divided into two categories. For example, 27% of singletons (13 of 35) but only 12% of the more frequent variants (2 of 15) were predicted to have functional effects based on SNAP2 (SNAP2 score > 0). The Mutpred predictions showed even larger differences between the rare and frequent variants, with 25% of the singletons (12 of 48) and none of the more frequent variants (0 of 17) predicted to have a high likelihood of potentially harmful effects (MutPred score ≥ 0.6; Fisher's exact test *p* = .027).

The distributions of estimated functional scores for interspecific comparisons were shifted toward lower values compared with the intraspecific analysis for both prediction methods, indicating that harmful nonsynonymous polymorphisms within populations rarely became fixed between species (Figure 3a,b). In contrast, the estimated effect of all possible variants for both stickleback species were strongly shifted toward high values, implying that a large proportion of potential variants have severe negative functional effect, resembling earlier results in mammals (Soares et al., 2013). No significant differences in mean functional scores were identified between freshwater and brackish water nine‐spined stickleback. However, based on MutPred predictions nine‐spined stickleback in brackish water environment showed slightly higher, albeit nonsignificant *(F*‐test *p* = .056), variance in pathogenicity scores compared with their freshwater conspecifics (Figure 3b).

Similar to earlier findings in mammals, the estimated selection functions for both prediction tools were consistent with the exponential decay (adjusted *r* ^2^ = .725–.992, all *p* < .002, Figure 3c,d). As expected, the steepest purifying selection gradient was observed for the interspecific comparison between the nine‐ and three‐spined sticklebacks. Within *P. pungitius,* the purifying selection gradient on the predicted functional scores (i.e., exponential decay) was slightly steeper in the freshwater environment than in the brackish water habitat for both prediction tools (Figure 3c,d).

## 4 Discussion

We hypothesized that the reduced energetic cost of osmoregulation in the brackish water habitat may result in the relaxation of selective constraints at the mitochondrial OXPHOS genes. In turn, this hypothesis predicts that harmful mutations at the mitochondrial OXPHOS genes linked to energy production may have less severe fitness consequences in brackish water habitats. However, the functional predictions did not provide support for osmoregulation‐dependent relaxation of purifying selection hypothesis at the mitochondrial OXPHOS genes in nine‐spined stickleback. Instead, an elevated ratio of nonsynonymous to synonymous variants observed in freshwater (*P*_n_ */P*_s_ = 0.549) compared with brackish water habitat (*P*_n_ */P*_s_ = 0.283) and slightly higher proportion of weakly deleterious mutations in freshwater habitat (δ = 84%, 95% CI: 72%–90% vs. δ = 68%, 95% CI: 52%–78%) supported the hypothesis that a reduced effective population size may drive the efficacy of purifying selection at mtDNA OXPHOS genes in nine‐spined stickleback. This finding is in line with analysis in mammals, which demonstrates more efficient purifying selection in species with large *N*_e_ (Popadin et al., 2007), as the dynamics of nearly neutral deleterious variants are primarily affected by random drift if their effects on fitness are <1/*N*_e_ for haploid loci (Kimura, 1962; Ohta, 1992).

On the other hand, it is important to keep in mind that the behaviour of the ratio of nonsynonymous to synonymous variants is radically different when it is used on segregating polymorphisms in contrast to interspecific data (Sawyer & Hartl, 1992). For example, Kryazhimskiy and Plotkin (2008) demonstrated that the *d*_N_/*d*_S_ ratio calculated based on polymorphisms within a population was not a monotonic function of the scaled selection coefficient, depended on the mutation rate and was relatively insensitive to the scaled selection coefficient. As a result, the observation of a *d*_N_/*d*_S_ = 0.1 is consistent with rather strong negative selection within a population, whereas this observation reflects only weak negative selection between divergent lineages (Kryazhimskiy & Plotkin, 2008). Furthermore, small shifts in the observed *d*_N_/*d*_S_ or *P*_n_/*P*_s_ ratio at low values may imply large differences in the strength of purifying selection between populations when the ratio is calculated based on polymorphism data (see Figure 1 in Kryazhimskiy & Plotkin, 2008 for *d*_N_/*d*_S_). Furthermore, the nine‐spined stickleback samples used in this study were collected from multiple populations showing different, albeit low, levels of genetic divergence (Table S5), which may further complicate the interpretation of the ratio of nonsynonymous to synonymous variants (Kryazhimskiy & Plotkin, 2008). Thus, further work is needed to confirm whether the higher proportion of nonsynonymous substitutions observed in the freshwater nine‐spined stickleback at mitochondrial OXPHOS genes is caused by less efficient purifying selection in small populations.

Over the last decade, increasing numbers of studies in humans have predicted the effects of nonsynonymous variants from exome and whole genome data (e.g., Wu et al., 2016). However, only recently have pathogenicity prediction tools been applied to evolutionary genetics in other organisms (Barson et al., 2015; Soares et al., 2013). Our study presents four lines of evidence demonstrating that the in silico characterization of the functional effects of nonsynonymous mitochondrial polymorphisms provides novel, evolutionary meaningful insights for a teleost fish. First, similar to mammals (Pereira et al., 2011; Soares et al., 2013), comparison of the predicted functional effect between the observed and all potential nonsynonymous polymorphisms in the nine‐spined stickleback revealed that a large majority of the predicted harmful amino acid variants have been eliminated by purifying selection. Second, a low proportion of the variants with strong predicted effects resulted in a steep purifying selection gradient on the predicted functional scores, which resembled earlier results in mammals (Pereira et al., 2011; Soares et al., 2013). Third, fixed interspecific nonsynonymous substitutions showed weaker predicted effects than nonsynonymous polymorphisms within species, which is consistent with the excess of weakly or moderately deleterious polymorphisms segregating within populations (Hasegawa et al., 1998; Nachman et al., 1994; Rand & Kann, 1996). Finally, rare variants showed an excess of predicted harmful effects, consistent with earlier findings in humans (Henn et al., 2016; Lohmueller, 2014; Subramanian, 2012).

Despite the improved performance of recent in silico prediction methods, identification of weakly to moderately harmful variants is not trivial and comparative studies have often showed considerable variation among different prediction tools (Hecht et al., 2015; Thusberg et al. 2011; Walters‐Sen et al. 2015). One concern is an overlap between training and evaluation datasets, implying that the in silico tools may result in overly optimistic predictions for known variants, but perform worse on novel variants or species (Grimm et al., 2015). Our results concur with these findings because the functional scores between the two prediction tools were correlated but at the same time showed considerable variation for specific variants. For example, we observed moderate correlations (*r*_S_ = .57–.58) between two prediction methods when all possible variants were compared within both *P. pungitius* and *G. aculeatus,* which most likely is related to congruent prediction of highly deleterious variants. On the other hand, SNAP2 and MutPred scores for observed fixed variants between *P. pungitius* and *G. aculeatus* were not correlated, which likely reflects the lack of strongly deleterious fixed substitutions between species. The variable outcomes of the prediction tools also highlight the value of large scale sequencing efforts to identify sites in the genome that are highly conserved. For example, recent analysis of over 10,000 human genomes enabled to the pinpointing conserved positions in the genome that are under strong purifying selection (Telenti et al., 2016). Finally, the potential problems with circularity of prediction tools (Grimm et al., 2015) call for further empirical experimental work to evaluate the actual fitness consequences of the observed variants. For example, comparison of the predicted effects of amino acid variants at the mitochondrial OXPHOS genes with the whole organism's performance, such as the standard metabolic rate (Bruneaux et al., 2014) or polarographic studies of oxygen consumption and spectrophotometric analyses of the mitochondrial respiratory chain enzymes (Barrientos, 2002), would allow an evaluation of the physiological effects of naturally occurring nonsynonymous changes (Brown, Lee, & Thorgaard, 2006). One of the first experimental studies that evaluated the functional consequences of the predicted amino acid effects found that only half of the supposedly deleterious mutations conferred clinical phenotypic effects in mice (Miosge et al., 2015). If these results based on evaluation of 33 missense mutations at essential immune genes can be generalized to the whole genomes, a large proportion of predicted deleterious mutations probably correspond to nearly neutral mutations with weak effects. Thus, evolutionary genetic inferences using functional prediction tools, such as presented here, are expected to provide valuable and complementary insights to experimental efforts that aim to validate the phenotypic effects of the observed amino acid variants.

In summary, this study demonstrated that weakly or moderately deleterious variants in the mitochondrial genome were common in wild teleost fish species, resembling earlier findings in *Drosophila*, mice, and humans (Hasegawa et al., 1998; Rand & Kann, 1996). The functional predictions provided weak support for the osmoregulation‐dependent relaxation of purifying selection hypothesis in brackish water environment. In contrast, elevated frequency of nonsynonymous polymorphisms in the freshwater environment indicated that the efficacy of purifying selection might be weaker in freshwater populations with low *N*_e_. However, screening larger number of mitogenomes preferably in multiple fish species is still needed to clarify the potential role of *N*_e_ and of the osmoregulatory energy budget on purifying selection at the OXPHOS genes. Similarly, experimental quantification of the osmoregulatory energy budgets at various salinities is necessary to better understand the potential relationships between purifying selection on the mitochondrial OXPHOS genes and osmoregulatory energy expenditure of freshwater, brackish water, and marine fish. Thus, mitochondrial energy production genes represent an excellent system to evaluate the functionality, pathogenicity, and habitat‐dependent fitness effects of nonsynonymous mutations, which will help unravel the processes that drive and limit evolution in natural populations (Ballard & Melvin, 2010).

## Ethics

Collection of specimens was conducted in accordance with national permit (58/2012 given to AV).

## Conflict of Interests

None declared.

## Authors’ Contributions

AV conceived the study and wrote the first draft of the manuscript. AV, MB, JS, and OT performed sampling and laboratory work. MO, JS, MB, OT, HM, and AV analyzed the data.
