# Supplementary material for: Prediction of harmful variants on mitochondrial genes: Test of habitat‐dependent and demographic effects in a euryhaline fish
Source: Ecol Evol. 2017 Apr 18;7(11):3826–35. doi: 10.1002/ece3.2989 (PMC5468147; doi:10.1002/ece3.2989)
Supplement: Supplementary file 1 [file ECE3-7-3826-s001.pdf]

Table S1. Primers used in a long-range PCR to amplify mitochondrial genome of nine-spined stickleback.

Name	*Sequence (5'-3')*
A_for_old	AGAGTAACCATGGGGGTTA
A_rev_old	ACTCCTGCTTGGATTTGCAC
B_for_old	CCCTTACCCACCGAGAGAG
B_rev_old	CCAACATGTTTGGGGTATGG
A_for_new	TGGAGCTGTGCCTGAAGTAA
A_rev_new	AGTGAGCTCTGGGGTTCAAC
B_for_new	CGAACTAGTTTGGGGTTGAG C
B_rev_new	GCCGAAAAGAAGTGTGGCTA

The primers used in the polymerase chain reaction (PCR) were designed with Primer3 (Untergasser *et al*. 2012) using the *P. pungitius* mitochondrial genome sequence from GenBank (accession number AB445130) as a template. Two primer pairs were designed, both producing a ~9 kb segments covering one half the mitochondrial genome with a ~200 bp overlap where the segments met. The primers were designed to anneal to highly conserved areas of the mitochondrial genome to minimize the risk of mismatches. When a sample failed to amplify one or both segments, we designed two new primer pairs and used these or a combination of original and new primers.

Untergasser A, Cutcutache I, Koressaar T, Ye J, Faircloth BC, Remm M, Rozen SG (2012) Primer3-new capabilities and interfaces. *Nucleic acids research* 40:108-115.

Table S2. Sample information and summary of the sequencing runs.

Sample ID	*Population*	*Habitat*	*Ion PGM™ Chip*	*Minimum coverage**	*Average coverage*	*Reads with barcode*	*No. fragments aligned*	*% aligned*	*No. of ambiguous nucleotides marked as "N"*	*% of ambiguous nucleotides marked as "N"*
jan001	Jäneda	freshwater	316	116	388.1	33461	32416	96.88	3	0.02
jan008	Jäneda	freshwater	316	64	200.9	17191	16561	96.34	0	0.00
jan028	Jäneda	freshwater	316	170	729.6	64692	59110	91.37	2	0.01
jan041	Jäneda	freshwater	316	245	640.5	56068	54073	96.44	0	0.00
jarle001	Järlepa	freshwater	316	86	369.3	31575	30116	95.38	0	0.00
jarle002	Järlepa	freshwater	316	243	733.5	63601	60642	95.35	0	0.00
jarle003	Järlepa	freshwater	316	168	507.7	48194	41664	86.45	0	0.00
jarle004	Järlepa	freshwater	316	162	775.1	83809	63137	75.33	0	0.00
kunda57	Kunda	brackish water	314	0	74.3	9967	7210	72.34	39	0.24
kunda65	Kunda	brackish water	314	0	23.3	2584	2163	83.71	96	0.59
kunda66	Kunda	brackish water	314	0	26.7	2967	2540	85.61	41	0.25
kunda75	Kunda	brackish water	314	0	25.0	2817	2344	83.21	120	0.73
kunda79	Kunda	brackish water	314	0	16.5	2078	1576	75.84	643	3.94
kundak1_1	Kunda	brackish water	316	27	274.0	23363	22667	97.02	0	0.00
kundak1_10	Kunda	brackish water	316	63	387.0	34244	32285	94.28	0	0.00
kundak1_6	Kunda	brackish water	316	149	643.5	55262	53242	96.34	1	0.01
kundak2_13	Kunda	brackish water	316	65	441.5	38362	36887	96.16	0	0.00
lobi74	Lobi	brackish water	314	0	17.9	2491	1672	67.12	293	1.79
lobi83	Lobi	brackish water	314	0	15.7	2083	1595	76.57	967	5.92
lobil1_10	Lobi	brackish water	316	62	347.1	29139	28214	96.83	0	0.00
lobil1_8	Lobi	brackish water	316	80	503.5	43153	41322	95.76	2	0.01
lobil2_21	Lobi	brackish water	316	24	293.8	24775	23907	96.50	0	0.00
lobil2_25	Lobi	brackish water	316	33	335.1	28874	27750	96.11	0	0.00
lobil2_8	Lobi	brackish water	316	59	427.6	36357	34867	95.90	0	0.00
modr52	Mõdriku	freshwater	314	0	13.1	2535	1285	50.69	1068	6.54
modr61	Mõdriku	freshwater	314	0	17.1	1831	1573	85.91	286	1.75
modr62	Mõdriku	freshwater	314	0	36.1	5105	3458	67.74	75	0.46
modr64	Mõdriku	freshwater	314	4	48.2	5175	4545	87.83	15	0.09
modrm1_11	Mõdriku	freshwater	316	131	780.0	75958	62735	82.59	10	0.06
mois001	Mõisalaht	brackish water	316	19	503.8	43450	42375	97.53	0	0.00
mois003	Mõisalaht	brackish water	316	74	513.3	42626	40998	96.18	0	0.00
mois190	Mõisalaht	brackish water	316	201	758.6	63259	60916	96.30	0	0.00
pid004	Pidula	freshwater	316	105	393.1	33858	32559	96.16	1	0.01
pid008	Pidula	freshwater	316	140	515.4	43535	41797	96.01	0	0.00
pid214	Pidula	freshwater	316	214	686.8	63321	56642	89.45	0	0.00
pid231	Pidula	freshwater	316	187	764.4	88830	62892	70.80	17	0.10
polul54	Põlula	freshwater	314	0	13.2	2063	1339	64.91	440	2.69
polul63	Põlula	freshwater	314	0	72.4	8167	6891	84.38	56	0.34
polul76	Põlula	freshwater	314	0	13.8	1591	1321	83.03	420	2.57
polul82	Põlula	freshwater	314	0	20.4	2379	2013	84.62	339	2.08
roop004	Roopa	brackish water	316	162	536.5	47515	45471	95.70	1	0.01
roop006	Roopa	brackish water	316	70	359.7	32284	29122	90.21	0	0.00
roop012	Roopa	brackish water	316	49	288.2	25819	23456	90.85	0	0.00
roop242	Roopa	brackish water	316	86	262.5	22900	21704	94.78	0	0.00
roop243	Roopa	brackish water	316	23	243.2	22555	19907	88.26	0	0.00
roop260	Roopa	brackish water	316	64	481.2	40828	39326	96.32	1	0.01
roop261	Roopa	brackish water	316	70	526.1	46230	43866	94.89	0	0.00
roop265	Roopa	brackish water	316	61	396.8	33851	32327	95.50	9	0.06
roop268	Roopa	brackish water	316	52	246.2	20944	20023	95.60	1	0.01
roos106	Roosna-Alliku	freshwater	316	112	746.8	100000	62528	62.53	0	0.00
roos112	Roosna-Alliku	freshwater	316	106	425.7	35947	34508	96.00	0	0.00
roos121	Roosna-Alliku	freshwater	316	96	297.9	25111	24232	96.50	5	0.03
roos131	Roosna-Alliku	freshwater	316	96	341.8	29919	28782	96.20	5	0.03
roos140	Roosna-Alliku	freshwater	316	40	172.6	17923	14099	78.66	1	0.01
sau017	Saulepi	brackish water	316	38	184.4	17290	15455	89.39	1	0.01
sau029	Saulepi	brackish water	316	71	335.7	31677	27123	85.62	3	0.02
seili4	Seili	brackish water	316	51	520.5	44359	41939	94.54	1	0.01

*The positions with coverage <5 reads were marked as "N".

Table S3. Summary statistics for 57 nine-spined stickleback mitogenomes.

Gene/ feature	*Habitat*	*length (nt)*	*S*	P_n	P_s	P_n/P_s	d_N/d_S	N_h	*h* (±SD)	π (±SD)	θ (±SD)
ATP6	freshwater	684	3	2	1	2.000	0.72	4	0.672 (±0.008)	0.00123 (±0.00017)	0.00121 (±0.00070)
	brackishwater	684	9	4	5	0.800	0.29	10	0.576 (±0.109)	0.00121 (±0.00030)	0.00371 (±0.00124)
	All	684	11	5	6	0.833	0.30	7	0.393 (±0.078)	0.00111 (±0.00026)	0.00313 (±0.00128)
ATP8	freshwater	168	1	1	0	n/a	n/a	2	0.095 (±0.084)	0.00057 (±0.00050)	0.00165 (±0.00165)
	brackishwater	168	4	2	2	1.000	0.30	5	0.253 (±0.104)	0.00159 (±0.00069)	0.00601 (±0.00300)
	All	168	5	3	2	1.500	0.45	4	0.103 (±0.055)	0.00219 (±0.00119)	0.01355 (±0.00782)
CO1	freshwater	1551	7	0	7	0.000	0.00	6	0.719 (±0.085)	0.00099 (±0.00018)	0.00124 (±0.00047)
	brackishwater	1551	17	2	15	0.133	0.05	16	0.860 (±0.049)	0.00106 (±0.00016)	0.00285 (±0.00071)
	All	1551	22	2	20	0.100	0.03	15	0.598 (±0.077)	0.00078 (±0.00014)	0.00290 (±0.00068)
CO2	freshwater	691	3	0	3	0.000	0.00	4	0.230 (±0.110)	0.00035 (±0.00017)	0.00115 (±0.00067)
	brackishwater	691	10	1	9	0.111	0.03	10	0.503 (±0.111)	0.00100 (±0.00028)	0.00386 (±0.00122)
	All	691	12	1	11	0.091	0.03	11	0.323 (±0.081)	0.00060 (±0.00017)	0.00370 (±0.00112)
CO3	freshwater	786	6	2	4	0.500	0.17	7	0.812 (±0.055)	0.00171 (±0.00023)	0.00210 (±0.00086)
	brackishwater	786	8	0	8	0.000	0.00	9	0.469 (±0.114)	0.00068 (±0.00019)	0.00259 (±0.00092)
	All	786	14	2	12	0.167	0.06	10	0.429 (±0.082)	0.00063 (±0.00014)	0.00258 (±0.00086)
CytB	freshwater	1141	10	4	6	0.667	0.23	9	0.857 (±0.042)	0.00185 (±0.00022)	0.00235 (±0.00074)
	brackishwater	1141	18	2	16	0.125	0.04	13	0.632 (±0.102)	0.00102 (±0.00025)	0.00397 (±0.00093)
	All	1141	26	6	20	0.300	0.10	20	0.749 (±0.004)	0.00142 (±0.00019)	0.00481 (±0.00096)
ND1	freshwater	975	7	1	6	0.167	0.06	6	0.500 (±0.121)	0.00092 (±0.00031)	0.00194 (±0.00073)
	brackishwater	975	20	6	14	0.429	0.15	16	0.851 (±0.057)	0.00185 (±0.00031)	0.00521 (±0.00116)
	All	975	23	6	17	0.353	0.12	16	0.555 (±0.006)	0.00100 (±0.00020)	0.00429 (±0.00098)
ND2	freshwater	1047	8	5	3	1.667	0.62	5	0.563 (±0.103)	0.00099 (±0.00033)	0.00216 (±0.00076)
	brackishwater	1047	20	7	13	0.538	0.20	15	0.759 (±0.089)	0.00137 (±0.00027)	0.00493 (±0.00110)
	All	1047	26	11	15	0.733	0.27	10	0.293 (±0.080)	0.00065 (±0.00020)	0.00400 (±0.00116)
ND3	freshwater	351	1	0	1	0.000	0.00	2	0.080 (±0.072)	0.00023 (±0.00021)	0.00077 (±0.00077)
	brackishwater	351	4	0	4	0.000	0.00	5	0.308 (±0.107)	0.00094 (±0.00036)	0.00290 (±0.00145)
	All	351	5	0	5	0.000	0.00	4	0.103 (±0.055)	0.00042 (±0.00023)	0.00259 (±0.00150)
ND4	freshwater	1381	12	6	6	1.000	0.35	10	0.867 (±0.048)	0.00175 (±0.00022)	0.00234 (±0.00067)
	brackishwater	1381	28	6	22	0.273	0.09	16	0.776 (±0.084)	0.00125 (±0.00026)	0.00443 (±0.00092)
	All	1381	39	11	28	0.393	0.14	24	0.803 (±0.054)	0.00158 (±0.00021)	0.00602 (±0.00102)
ND4L	freshwater	297	0	0	0	n/a	n/a	1	0	0	0
	brackishwater	297	4	1	3	0.333	0.12	5	0.299 (±0.105)	0.00114 (±0.00043)	0.00360 (±0.00180)
	All	297	4	1	3	0.333	0.12	5	0.169 (±0.067)	0.00078 (±0.00032)	0.00389 (±0.00194)
ND5	freshwater	1839	18	5	13	0.385	0.12	10	0.880 (±0.043)	0.0014 (±0.00026)	0.00264 (±0.00062)
	brackishwater	1839	33	7	26	0.269	0.09	22	0.917 (±0.046)	0.00113 (±0.00016)	0.00429 (±0.00077)
	All	1839	49	12	37	0.324	0.10	31	0.895 (±0.036)	0.00122 (±0.00015)	0.00547 (±0.00082)
ND6	freshwater	522	3	2	1	2.000	0.77	4	0.410 (±0.111)	0.00101 (±0.00031)	0.00183 (±0.00105)
	brackishwater	522	11	3	8	0.375	0.14	8	0.579 (±0.104)	0.00184 (±0.00055)	0.00498 (±0.00157)
	All	522	14	5	9	0.556	0.21	6	0.169 (±0.067)	0.00069 (±0.00033)	0.00425 (±0.00161)
All genes	freshwater	11412	79	28	51	0.549	0.188	14	0.923 (±0.033)	0.00086 (±0.00011)	0.00122 (±0.00018)
	brackishwater	11412	186	41	145	0.283	0.097	31	1.000 (±0.008)	0.00110 (±0.00009)	0.00400 (±0.00031)
	All	11412	248	65	183	0.355	0.12	43	0.979 (±0.010)	0.00100 (±0.00008)	0.00424 (±0.00031)
rRNA	freshwater	2635	19					9	0.794 (±0.067)	0.00080 (±0.00015)	0.00153 (±0.00039)
	brackishwater	2635	28					22	0.920 (±0.043)	0.00078 (±0.00010)	0.00252 (±0.00053)
	All	2635	46					30	0.873 (±0.041)	0.00081 (±0.00010)	0.00334 (±0.00053)
tRNA	freshwater	1542	7					3	0.151 (±0.093)	0.00026 (±0.00019)	0.00090 (±0.00045)
	brackishwater	1542	10					7	0.355 (±0.110)	0.00041 (±0.00015)	0.00140 (±0.00053)
	All	1542	17					7	0.201 (±0.071)	0.00029 (±0.00012)	0.00182 (±0.00061)
D-loop	freshwater	672	10					8	0.497 (±0.108)	0.00117 (±0.00032)	0.00369 (±0.00105)
	brackishwater	672	10					3	0.517 (±0.087)	0.00142 (±0.00043)	0.00209 (±0.00131)
	All	672	15					8	0.445 (±0.078)	0.00116 (±0.00026)	0.00358 (±0.00127)
Total	freshwater	16335	115					19	0.969 (±0.020)	0.00089 (±0.00009)	0.00140 (±0.00016)
	brackishwater	16335	236					31	1.000 (±0.008)	0.00099 (±0.00007)	0.00353 (±0.00025)
	All	16335	330					48	0.991 (±0.006)	0.00095 (±0.00007)	0.00398 (±0.00025)

S - the number of segregating sites, P_n - the number of nonsynonymous polymorphisms, P_s - the number of synonymous polymorphisms, d_N/d_S ratio, N_h - the number of haplotypes, *h* - haplotype diversity, π - nucleotide diversity per site and θ per site.

Table S4. Summary of variable amino acid sites found within each protein-coding gene and predicted functional (SNAP2) and patogenecity (MutPred) scores observed among *P. pungitius* samples.

Protein	*Amino acid variant*			*Functional score*		*Sample ID with observed alternative amino acid in a protein*	*Habitat*	
	Pos	*Ref*	*Alt*	*SNAP2*	*MutPred*			
ATP6	17	I	V	-38	0.516	mois190		brackish water
ATP6	21	A	T	-36	0.474	pid004, pid214, sau029	freshwater	brackish water
ATP6	36	A	T	-58	0.321	lobi74		brackish water
ATP6	101	I	V	-47	0.48	jan001, jan008, jan041, roos112	freshwater	
ATP6	144	I	V	-56	0.347	kunda75		brackish water
ATP8	19	V	I	-19	0.463	jan028	freshwater	
ATP8	32	V	M	**6**	0.327	lobi74		brackish water
ATP8	38	T	A	**7**	0.424	kundak1_6		brackish water
CO1	83	I	V	-94	0.488	kundak1_10, lobil1_8		brackish water
CO1	515	S	L	-73	0.363	roop261		brackish water
CO2	225	R	Q	-69	0.327	kunda75		brackish water
CO3	40	T	A	**16**	0.245	jan001, jan008, jan041, roos112	freshwater	
CO3	107	A	T	-46	0.53	pid004, pid214	freshwater	
CytB	19	V	I	-82	0.501	jarle001	freshwater	
CytB	112	T	M	**34**	0.595	jan001, jan008, jan041	freshwater	
CytB	192	A	T	-54	0.504	modr52	freshwater	
CytB	300	V	I	-69	0.55	modr61	freshwater	
CytB	372	V	L	-87	0.287	roop012		brackish water
CytB	377	L	I	-66	0.231	roop243		brackish water
ND1	2	I	T	-94	0.29	lobi74		brackish water
ND1	11	N	S	-37	**0.626**	roop268		brackish water
ND1	80	A	T	-71	0.599	pid004, pid214, sau029	freshwater	brackish water
ND1	145	T	A	**41**	**0.682**	roop006		brackish water
ND1	227	A	T	-7	**0.664**	roop260		brackish water
ND1	254	I	T	-47	0.386	kunda75		brackish water
ND2	56	T	A	-72	**0.6**	kundal1_1		brackish water
ND2	90	T	I	**12**	0.399	roop005		brackish water
ND2	153	V	I	-46	0.506	roop265		brackish water
ND2	243	T	I	-3	0.415	jan028	freshwater	
ND2	283	F	L	-74	0.462	roos121	freshwater	
ND2	288	S	N	**50**	**0.754**	kunda57		brackish water
ND2	308	V	M	-47	0.42	kundak1_10		brackish water
ND2	310	A	T	-23	0.426	pid008	freshwater	
ND2	317	P	Q	-58	0.262	pid008, kundak1_6	freshwater	brackish water
ND2	331	A	T	-47	0.501	pid008	freshwater	
ND2	336	L	M	-1	**0.75**	roop260		brackish water
ND4	20	T	I	-32	0.368	pid008	freshwater	
ND4	25	S	P	-48	0.349	polul54, polul63, polul76, polul82	freshwater	
ND4	42	F	L	-64	0.477	modr61, modr62, modr64, polul54, polul63, polul76, polul82	freshwater	
ND4	86	A	T	-82	0.336	lobil2_25		brackish water
ND4	87	S	G	-83	0.289	kundak1_6, pid008	freshwater	brackish water
ND4	90	Y	F	-80	0.292	lobi74		brackish water
ND4	135	T	N	**50**	**0.609**	roop242		brackish water
ND4	231	I	V	-93	**0.636**	roop265		brackish water
ND4	399	A	P	**52**	0.476	modr52	freshwater	
ND4	439	A	T	-86	0.397	roop265		brackish water
ND4	450	L	F	-67	0.417	modr61, modr62, modr64, polul54, polul63, polul76, polul82	freshwater	
ND4L	24	T	I	**52**	0.528	kunda66		brackish water
ND5	7	V	I	-71	0.434	modr61, modr62, modr64	freshwater	
ND5	31	N	S	-84	0.373	mois003		brackish water
ND5	58	M	V	-65	0.336	pid008	freshwater	
ND5	116	D	N	**57**	**0.856**	lobil1_10		brackish water
ND5	182	I	V	-96	0.581	roop261		brackish water
ND5	276	N	D	**47**	**0.705**	roop268		brackish water
ND5	400	A	T	-75	0.584	modr61, modr62, modr64	freshwater	
ND5	460	I	V	-40	**0.608**	roop260		brackish water
ND5	526	P	S	-26	0.179	lobil2_25		brackish water
ND5	559	T	A	-41	0.252	polul54, polul63, polul76, polul82	freshwater	
ND5	583	T	S	-58	0.427	roop004		brackish water
ND5	602	T	A	**50**	**0.77**	roos140	freshwater	
ND6	4	V	F	-58	0.362	kunda65		brackish water
ND6	91	M	T	**19**	**0.695**	kunda79		brackish water
ND6	101	V	M	-46	0.576	kunda65, kundak2_13, lobi83, mois190, seili4		brackish water
ND6	115	V	A	**15**	0.404	jan041	freshwater	
ND6	117	A	S	-35	0.399	jarle001, jarle002, jarle003, jarle004	freshwater	

Pos - amino acid position is based on the translated sequence of each gene. Ref - reference amino acid, Alt - alternative amino acid.

Variants with high likelihood of functional effect are highlited in bold (SNAP2 score>0; Mutpred score >0.6).

Table S5. Pairwise genetic distances (F_{ST}) among nine-spined stickleback samples based on 9 microsatellite loci (below diagonal) and their significance (above diagonal).

		Freshwater					Brackish water				
		Jäneda	Mõdriku	Põlula	Pidula	Roosna-Alliku	Kunda	Lobi	Mõisalaht	Roopa	Saulepi
Freshwater	Jäneda	-	*	*	*	*	*	*	*	*	*
	Mõdriku	0.3112	-	*	*	*	*	*	*	*	*
	Põlula	0.2724	0.4067	-	*	*	*	*	*	*	*
	Pidula	0.1364	0.2296	0.2747	-	*	*	*	*	*	*
	Roosna-Alliku	0.0587	0.2857	0.2609	0.0980	-	*	*	*	*	*
Brackish water	Kunda	0.0965	0.2279	0.2046	0.0718	0.0754	-	NS	NS	*	*
	Lobi	0.0949	0.2130	0.2085	0.0603	0.0848	0.0033	-	NS	NS	NS
	Mõisalaht	0.0953	0.2170	0.2222	0.0668	0.0845	0.0113	0.0061	-	NS	NS
	Roopa	0.0992	0.2049	0.2051	0.0657	0.0788	0.0128	0.0084	-0.0009	-	-
	Saulepi	0.0957	0.2157	0.2203	0.0576	0.0680	0.0097	0.0043	-0.0046	-0.0053	0.0000

*P < 0.05, NS = nonsignificant

Genetic differentiation between brackish water and freshwater populations was estimated using Weir & Cockerham's estimator of F_{ST} as implemented in the FSTAT program (Goudet 1995). The significance of F_{ST} estimates were estimated using 1000 permutations and multiple testing correction (Bonferroni) was applied at nominal level of P =0.05.
